# Addison's disease in antiphospholipid syndrome: A rare complication

**DOI:** 10.1002/ccr3.4107

**Published:** 2021-05-19

**Authors:** Farzaneh Yazdi, Mohammad Reza Shakibi, Hanieh Mirkamali, Amir Baniasad

**Affiliations:** ^1^ Neuroscience Research Center Institute of Neuropharmacology Kerman University of Medical Sciences Kerman Iran; ^2^ Endocrinology and Metabolism Research Center Institute of Basic and Clinical Physiology Science Kerman University of Medical Sciences Kerman Iran; ^3^ Student Research Committee Kerman University of Medical Sciences Kerman Iran

**Keywords:** Addison's disease, antiphospholipid syndrome, thrombosis

## Abstract

The association of APS and AI is rare, but it is very important, and in cases where there is an evidence in favor of the association of these two diseases, due to the importance of early treatment of both diseases, further evaluation is necessary.

## Graphical abstract

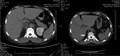

## 1 INTRODUCTION

A patient with Addison's disease and limb thrombosis was admitted due to abdominal pain. He was diagnosed with renal and inferior vena cava thrombosis while tested positive for Antiphospholipid syndrome (APS) and lupus. The early diagnosis of APS associated with Adrenal insufficiency is critical and requires a subtle examination.

Antiphospholipid syndrome is a systemic disease characterized by arterial and venous thrombotic events. This syndrome can be manifested initially (primary APS) or in association with other autoimmune diseases such as lupus erythematosus (secondary APS).^1^ Addison's disease (AD) is a rare endocrine disorder that affects men and women equally.^2^ AD has a variety of causes from infection to autoimmune diseases.^3^ AD is a rare APS complication, although it is the most common endocrine manifestation of the APS.^4^

In a study of 86 patients with Addison's disease and antiphospholipid syndromes, in about one‐third of the patients with the antiphospholipid syndrome, early onset of Addison's syndrome was reported. Accompanying Addison's disease and antiphospholipid disease can have a variety of causes, the most common one is the hemorrhage in the adrenal glands.^5^

We report a case of AD in a male patient with APS, probably autoimmune, without any evidence of adrenal hemorrhage or infarction.

## 2 CASE HISTORY

A 34‐years‐old patient with a 2‐year history of Adrenal insufficiency (AI) was referred to our hospital. The anticoagulant treatment of the patient had been started from 1 month before referral, following pain and swelling of the right lower extremity in another hospital diagnosed with deep vein thrombosis (DVT). The patient has also undergone a fasciotomy at the same time as being diagnosed with compartment syndrome. The patient was referred to our tertiary care hospital with a complaint of severe generalized abdominal pain. The patient's pain started 2 months ago, was persistent, gets worse with food intake, and did not radiate anywhere.

The patient did not have nausea, vomiting, diarrhea, constipation, blood in the stool, or urinary symptoms. The patient's lower extremity edema that developed about a month ago, had spread to the femur. The patient had no history of cough, hemoptysis, chest pain, or fever. Adrenal insufficiency of the patient had been diagnosed 2 years ago following signs of weight loss, buccal hyperpigmentation, orthostatic hypotension, and laboratory findings of sodium 126 mEq/L, potassium 6 mEq/L, cortisol 8 AM low serum level, and adrenocorticotropic hormone (ACTH) 492 (normal range 0‐11 pmol/L). The patient was treated with warfarin 5 mg daily, fludrocortisone 0.1 mg daily, and dexamethasone 0.5 mg every 12 hours. The patient's mother had a history of Rheumatoid arthritis.

The patient had a normal vital sign on physical examination (Blood Pressure: 110/80 mm Hg, Pulse Rate: 86/min, Temperature: 37.3°C, Respiratory Rate: 18/min, Oxygen saturation at room air: 92%). The patient had hyperpigmentation in the buccal mucosa, mild generalized tenderness in the abdomen, right lower limb swelling with pitting edema, and tenderness with a difference in size above 3 cm compared to the left lower limb. In the laboratory data, the patient had a normocytic anemia (Hemoglobin [Hb] = 12.5 [g/dL], Mean corpuscular volume [MCV] = 82 [FL]), thrombocytopenia (Platelets = 77 [×10^9^/L]) and an increase in erythrocyte sedimentation rate (ESR) (117 m.m.) and C‐reactive protein (CRP) (12) (Table 1).

**TABLE 1:** The laboratory data of the patient

Measure	Result	Reference range
WBC (×10^9^/L)	5.3	4‐10
Hb (g/dL)	12.4	13.5‐18
MCV (FL)	82	76‐96
Platelets (×10^9^/L)	77	150‐450
ESR (m.m.)	117	–
CRP	12	<6
urea (mg/dL)	40	17‐43
Cr (mg/dL)	1.1	0.7‐1.4
Na (mEq/L)	136	135‐140
K (mEq/L)	4.6	3.8‐5
AST (units/L)	62	<37
ALT (units/L)	46	<41
ALP (units/L)	168	80‐306
Total bilirubin (mg/dL)	1.7	1‐1.2
Direct bilirubin (μmol/L)	0.5	0.1‐0.3
PT (s)	15	–
PTT (s)	40	35‐40
INR	1.1	–
HBs‐Ag (Abs)	0.01	<0.218
HCV‐Ab (Abs)	0.01	<0.2
HIV‐Ab	0.01	<0.25
urinary protein	2+	–
24‐h urine protein (mg/24 h)	2430	–
Rheumatologic tests		
C3 (mg/dL)	145.7	90‐180
C4 (mg/dL)	25.09	10‐40
Anti‐CCP (AU/mL)	0.987	<12
Anticardiolipin IgG	≥ 100	<12
Anticardiolipin IgM	5.4	<12
B2‐glycoprotein Ab (IgG) (AU/mL)	130.2	<12
B2‐glycoprotein Ab (IgM) (AU/mL)	<3	<12
ANA (IU/mL)	392.23	<23
Anti‐DsDNA (IU/mL)	97.51	<12
C‐ANCA (AU/mL)	8.6	<12
P‐ANCA (AU/mL)	3.3	<12

Abbreviations: ALP, Alkaline phosphatase; ALT, Alanine aminotransferase; ANA, Antinuclear antibody; AST, Aspartate Aminotransferase; C‐ANCA, Cytoplasmic antineutrophil cytoplasmic antibodies; Cr, Creatinine; CRP, C‐reactive protein; ESR, Erythrocyte sedimentation rate; Hb, Hemoglobin; INR, International normalized ratio; MCV, Mean corpuscular volume; P‐ANCA, Perinuclear antineutrophil cytoplasmic antibodies; PT, Prothrombin time; PTT, Partial thromboplastin time; WBC, White blood cells.

In Abdominal and Pelvic ultrasound, the patient's liver was normal in size and echogenicity, and the gallbladder and bile ducts inside and outside the liver were normal. Portal hepatic veins were normal. The kidneys and spleen were normal in size and parenchyma. In Doppler ultrasound of the right lower‐limb manifestations of extensive thrombosis were observed in the tibial veins, popliteal vein, and semi‐distal femoral vein, and an extensive thrombosis was observed from the renal vein to the intrahepatic parts of (inferior vena cava) IVC.

**FIGURE 1:**
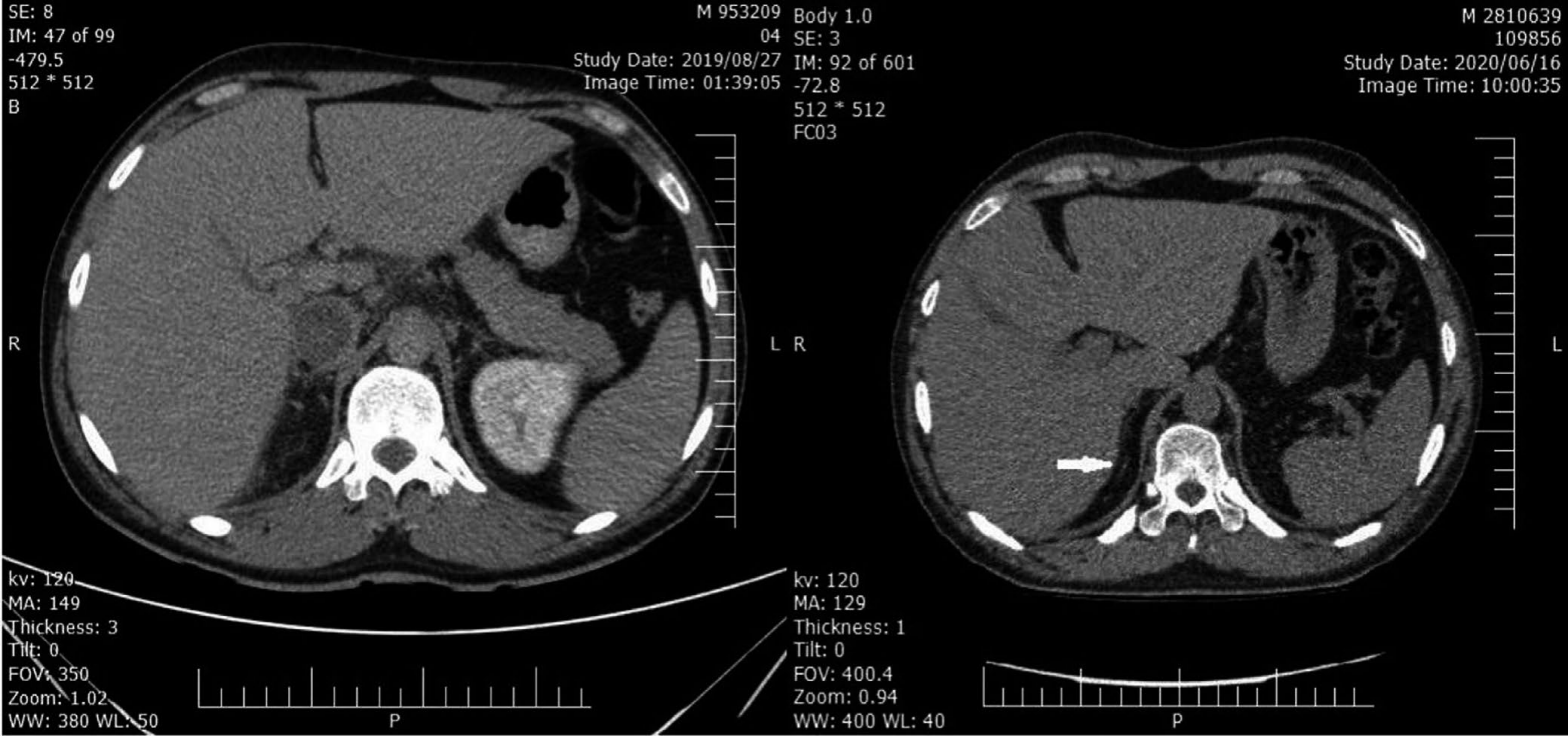
Right adrenal atrophy was seen on the patient's initial abdominal CT scan (left) and 9 mo later (right). On both CT scans, the left adrenal gland was not observed, probably due to severe atrophy

In the spiral CT scan of the abdomen and pelvis, extensive thrombosis within the IVC at renal level upward to the upper intrahepatic part of IVC was observed. Also, there were multiple significant para‐aortic, retrocaval, mesenteric lymphadenopathies. Spleen, pancreas, and kidneys were normal in size and with enhancement patterns. The right adrenal gland was atrophic. The left adrenal gland was not visible, which was probably due to its severe atrophy (Figure 1). In echocardiography, the ejection fraction (EF) was 60% and the pulmonary artery pressure (PAP) was 35 (mm Hg).

The patient had positive anticardiolipin antibody (IgG), B2‐glycoprotein antibody (IgG), antinuclear antibodies (ANA), and antidouble‐stranded DNA (Anti‐dsDNA) in rheumatologic lab tests. Other Rheumatologic laboratory tests are given in Table 1.

The patient had proteinuria 2+ in urinalysis and 2430 mg of protein in 24‐hour urine and while he was a candidate for kidney biopsy to rule out lupus nephritis, due to the patient's condition, it was not possible to discontinue the patient's anticoagulant, and so the biopsy was not performed.

After establishment of the association of APS and AI, treatment with tablet prednisolone (1 mg/kg/day), injection cyclophosphamide (1 gr/month), tablet hydroxychloroquine (200 mg/day), tablet losartan (25 mg/day), tablet calcitriol (0.25 mg/day), tablet ASA (80 mg/day), tablet atorvastatin (20 mg/day), and warfarin (5 mg/day) were administered to the patient. The patient was discharged with the advice of monthly follow‐ups.

Cyclophosphamide (1 gr/month) was continued for 6 months and then CellCept (2 gr/daily) was started for the patient. We plan to continue CellCept for 2 years and then decide whether to continue or change the patient's immunosuppressive treatment based on the patient's clinical and paraclinical findings in the follow‐up visits. The Hydroxychloroquine was continued at the same dose for the patient. After 6 weeks of administration of prednisolone (1 mg/kg/day), it was tapered slowly to 7.5 mg/day and fludrocortisone (0.1 mg daily) was started again.

In the follow‐up of the patient, after 9 months on the abdominal CT scan, severe right adrenal atrophy was observed again, and the left adrenal gland was not seen (Figure 1).

## 3 DISCUSSION

Primary adrenal insufficiency (PAI) is a life‐threatening disease characterized by the inability of the adrenal cortex to secrete glucocorticoids or mineralocorticoids (1). Adrenal insufficiency was primary in our patient considering low serum levels of cortisol at 8 AM and high ACTH. The most common etiologies of this disease are autoimmune (80%‐90% of cases) and other etiologies are drugs, congenital adrenal hyperplasia, infectious or infiltrative disorders, adrenal metastases, Adrenoleukodystrophy, and in rare cases hemorrhage or infarction caused by APS.^6^, ^7^

Numerous organs, including the adrenal glands, may be affected by APS. Antiphospholipid syndrome is an autoimmune systemic disorder that leads to arterial and venous thrombosis, pregnancy morbidity, and loss. Paraclinical findings in these patients show an increase in the antiphospholipid antibodies.^8^ In a study by Cervera et al^9^ of 1000 patients with primary or secondary APS, 4 patients (0.4%) had Addison's syndrome. There are several hypotheses about the mechanisms for how adrenal involvement occurs in APS. In the first mechanism, specific vascularization of the adrenal glands is responsible for the imbalance between a rich arterial supply and limited venous drainage, which leads to abrupt arterial‐capillary network transition and causes venous thrombosis, followed by postinfarction hemorrhage.^6^, ^10^

In another important mechanism, high levels of membrane lysobisphosphatidic acid in zona fasciculate act as a target for antiphospholipid antibodies, leading to apoptosis and release of lysosomal proteinases, resulting in activated endothelial cells and microthrombosis.^10^

In a study by Lee et. al., 77 patients with AI and APS were examined. In 18 patients (23%), APS was diagnosed before AI, in 46 patients (60%) APS and AI diagnosed at the same time, and in 13 patients (17%) APS was diagnosed after AI.^6^ In a study by Espinosa et. al. on 86 patients with adrenal involvement and APS, 31 patients (36%) had the initial manifestation of APS disease.^5^ Oliveira et al reported a 36‐year‐old female known case of APS with multiple thrombotic events, including spontaneous abortion. After the third abortion, an adrenal hematoma was observed in abdominal CT but misdiagnosed and a few weeks later, the patient presented to the emergency department with the manifestations of adrenal insufficiency.^7^

Antiphospholipid syndrome can be seen primarily or in association with other autoimmune diseases, including lupus.^7^ According to the positive clinical and serological findings, the cause of APS in our patient is probably lupus, which can also explain the patient's proteinuria. To evaluate the patient for lupus nephritis as a cause of proteinuria, a kidney biopsy was considered for the patient, which was not performed due to the impossibility of discontinuing anticoagulants in the patient.

In our patient, the diagnosis of APS was done after AI and AI was initially diagnosed. Due to the rarity of AI in APS patients and the nonspecific nature of the manifestations of this complication, more attention should be taken to diagnose this complication.^7^ And if a patient with APS presenting with symptoms such as abdominal complaints, weakness, or asthenia, the patient's blood pressure, and serum sodium and potassium levels should be carefully evaluated, and additional diagnostic tests and treatment should be performed at the appropriate time for the patient.^7^

On the other hand, in patients whose etiology of adrenal insufficiency is unknown, APS tests should be performed even in the absence of thrombolytic disorders.^7^ Anticoagulation is still the standard treatment for APS patients. The purpose of international normalized ratio (INR) in these patients is yet debated. It is generally recommended in APS patients with the first episode of unprovoked venous thrombosis, INR 2‐3, and with the first episode of arterial thrombosis INR 2‐3 or INR 3‐4.^11^

In the study of Chu et al, it is recommended adjusting INR between 2 and 3 to prevent hemorrhage.^10^ In the treatment of patients with AI, treatment with glucocorticoids should be initiated, and after confirmation of aldosterone deficiency, the replacement of mineralocorticoids with fludrocortisone should be added.^7^

## 4 CONCLUSION

Considering that the association of APS and AI is rare, its diagnosis requires a high clinical suspicion, and regarding the importance of early diagnosis and treatment for these patients, screening for specific APS antibodies in patients with adrenal insufficiency should be performed. Screening is also necessary in terms of the hypoadrenalism in APS patients, especially in cases where there is evidence of the association between the two diseases.

## CONFLICT OF INTEREST

The authors acknowledge that there is no conflict of interest.

## AUTHOR CONTRIBUTIONS

Farzaneh Yazdi: served as a primary rheumatologist, involved in the patient's treatment, and wrote and edited the case report. Mohammad Reza Shakibi: served as a secondary rheumatologist, involved in patient's treatment, and revised the case report. Hanieh Mirkamali: wrote and edited the case report. Amir Baniasad: collaborated in patient treatment, wrote the initial manuscript, edited the final document, and submitted the article.

## ETHICAL APPROVAL

The informed consent of the patient has been obtained and the therapy procedure has been approved by the “Iran National Committee for Ethics in Biomedical Research” (http://ethics.research.ac.ir/IndexEn.php, no.: IR.KMU.AH.REC.1399.051).

## Data Availability

The data that support the findings of this study are available from the corresponding author upon reasonable request.
